# Evaluation of commercial devices for patient specific QA of stereotactic radiotherapy plans

**DOI:** 10.1002/acm2.14009

**Published:** 2023-05-09

**Authors:** Shands James, Ahmad Al‐Basheer, Eric Elder, Chulhaeng Huh, Christopher Ackerman, John Barrett, Russell Hamilton, Farshad Mostafaei

**Affiliations:** ^1^ Department of Radiation Oncology Medical College of Georgia, Augusta University Augusta Georgia USA; ^2^ Nuclear/Radiological Engineering/Medical Physics Department Georgia Institute of Technology Atlanta Georgia USA; ^3^ Department of Radiation Oncology Emory University School of Medicine Atlanta Georgia USA; ^4^ Department of Radiation Oncology University of Arizona Tucson Arizona USA

**Keywords:** diodes, electronic portal imaging device, quality assurance, radiochromic film, stereotactic body radiation therapy, stereotactic radiosurgery

## Abstract

Stereotactic radiotherapy (SRT) methods have become common for the treatment of small tumors in various parts of the body. Small field dosimetry has a unique set of challenges when it comes to the pre‐treatment validation of a radiotherapy plan that involves film dosimetry or high‐resolution detectors. Comparison of commercial quality assurance (QA) devices to the film dosimetry method for pre‐treatment evaluation of stereotactic radiosurgery (SRS), fractionated SRT, and stereotactic body radiation therapy treatment plans have been evaluated in this study. Forty stereotactic QA plans were measured using EBT‐XD film, IBA Matrixx Resolution, SNC ArcCHECK, Varian aS1200 EPID, SNC SRS MapCHECK, and IBA myQA SRS. The results of the commercial devices are compared to the EBT‐XD film dosimetry results for each gamma criteria. Treatment plan characteristics such as modulation factor and target volume were investigated for correlation with the passing rates. It was found that all detectors have greater than 95% passing rates at 3%/3 mm. Passing rates decrease rapidly for ArcCHECK and the Matrixx as criteria became more strict. In contrast, EBT‐XD film, SNC SRS MapCHECK, and IBA myQA SRS passing rates do not decline as rapidly when compared to Matrix Resolution, ArcCHECK, and the EPID. EBT‐XD film, SNC SRS MapCHECK, and IBA myQA SRS maintain greater than 90% passing rate at 2%/1 mm and greater than 80% at 1%/1 mm. Additionally, the ability of these devices to detect changes in dose distribution due to MLC positioning errors was investigated. Ten VMAT SBRT/SRS treatment plans were created with 6 MV FFF or 10 MV FFF beam energies using Eclipse 15.6. A MATLAB script was used to create two MLC positioning error scenarios from the original treatment plan. It was found that errors in MLC positioning were most reliably detected at 2%/1 mm for high‐resolution detectors and that lower‐resolution detectors did not consistently detect MLC positioning errors.

## 1 INTRODUCTION

Stereotactic radiosurgery (SRS) was first proposed by Lars Leksell, a Swedish neurosurgeon, in 1951 as a means of treating inoperable brain lesions. Leksell utilized a frame that attached to the patients head to immobilize the patient in addition to its role in target localization. Leksell experimented with using x‐rays, gamma rays from Co‐60, and proton beams for his SRS treatments.^1^, ^2^, ^3^ Later, researchers such as Frederico Colombo, Ken Winston, and Wendell Lutz proposed methods of SRS using a linear accelerator using rotating arcs to deliver dose to the target. Initially, SRS was delivered in a single fraction, but more recently 1 to 5 fractions have been used depending on the size and pathology of the lesion.^4^, ^5^, ^6^

In 1995, Hamilton et al. published a paper detailing their experience with extra‐cranial SRS.^7^ They treated spinal lesions that had failed previous conventional radiotherapy to 8−10 gray in a single fraction. This extra‐cranial SRS later became known as stereotactic body radiotherapy (SBRT). Similarly, SBRT delivered high doses of radiation to small, well‐defined targets in 1 to 5 treatments. SBRT expands the theory of SRS to other sites of the body beyond the central nervous system and spine. However, the introduction of different treatment sites added treatment complexities due to target motion and heterogeneous target areas that made dose calculations more difficult.^3^, ^7^

Stereotactic radiotherapy (SRT) techniques such as SRS and SBRT involve delivering a high dose of radiation to a small target in a small number of fractions. These plans are designed to be highly conformal and have heterogeneous dose distributions with steep fall off gradients. Delivering a large amount of dose in a hypo‐fractionated setting will result in a high degree of tumor control. However, the specific radiobiological mechanisms behind this effect are poorly understood although the effect can be predicted using the linear quadratic formula for cell survival curves.^3^, ^8^

In modern radiotherapy, linear accelerator (LINAC)‐based SRT can take advantage of flattening filter free (FFF) beams and volume modulated arc therapy (VMAT) to produce complex dose distributions that can be delivered in a short period of time with the goal of sparing normal tissue. The small number of fractions means the consequences of a geometric miss of the target could have a significant impact on tumor control. Therefore, treatment delivery of LINAC‐based SRT plans demand a high degree of precision in target localization and LINAC mechanical and dosimetric tolerances. For example, the typical isocenter stability tolerance for a LINAC performing stereotactic treatments is 1 mm.^9^

SRT methods have become common for the treatment of small tumors in various parts of the body. Pre‐treatment quality assurance (QA) of the treatment plan is an essential part of a patient's course of treatment. The characteristics of a stereotactic treatment plan and the limitations of a LINAC mentioned above demand an appropriate detector that is capable of accurately measuring the dose distribution. Small field dosimetry has a unique set of challenges such as the loss of lateral electronic equilibrium, undersampling, or volume‐averaging with detectors with volumes approaching the size of the field.^9^, ^10^ When it comes to the validation of a treatment plan for use on a patient that involve film dosimetry or other high‐resolution detectors. Film dosimetry is a cost‐effective method that provides superior spatial resolution in comparison to ion chamber matrices and high‐resolution diode arrays. However, film dosimetry is work and time intensive. Some alternatives to film dosimetry are devices such as the IBA Matrixx Resolution (IBA Dosimetry, Schwarzenbruck, Germany), Sun Nuclear Corporation (SNC) ArcCHECK (Sun Nuclear, Melbourne, FL), Varian aS1200 electronic portal imaging device (EPID) (Varian, Palo Alto, CA), SNC SRS MapCHECK (Sun Nuclear, Melbourne, FL), and IBA myQA SRS (IBA Dosimetry, Schwarzenbruck, Germany). Fortunately, patient‐specific QA devices have advanced in recent years in their design and in the software used to analyze measured dose distributions. The goal of this study is to compare the performance of these commercial QA devices to the film dosimetry method for pre‐treatment evaluation of SRS, fractionated stereotactic radiotherapy (FSRT), and SBRT treatment plans.

## 2 MATERIALS AND METHODS

### 2.1 Plan selection

Forty stereotactic treatment plans were selected with varying target volumes for this study. All treatment plans were created in Eclipse 15.6 treatment planning system (TPS) using intensity modulated radiotherapy (IMRT) or volumetric modulated arc therapy (VMAT) delivery techniques. Additionally, beam energies used during planning included 6 MV, 10 MV, 6 MV FFF, and 10 MV FFF. Target volume sizes ranged from 0.43 cm^3^ to 161.13 cm^3^ with a mean volume of 35.81 cm^3^. The 40 plans included 8 lungs, 8 abdomens, 11 cranial SRS or FSRT, 6 pelvises, 5 bone metastases, one head and neck nodule, and one chest wall metastases. Plan modulation factors (MU/cGy) ranged from 2.08 to 5.15 with a mean of 3.51. All 40 plans were delivered onto six different patient specific QA (PSQA) devices: EBT‐XD film (Ashland, Wilmington, DE), IBA Matrixx Resolution (IBA Dosimetry, Schwarzenbruck, Germany), SNC ArcCHECK (Sun Nuclear, Melbourne, FL), Varian aS1200 EPID (Varian, Palo Alto, CA), SNC SRS MapCHECK (Sun Nuclear, Melbourne, FL), and IBA myQA SRS (IBA Dosimetry, Schwarzenbruck, Germany).

### 2.2 Detectors

#### 2.2.1 EBT‐XD film

A calibration curve was created from 0 to 2800 cGy using 23 data points. The dose was delivered to each film using 6 MV energy at 5 cm depth and 10 cm of backscatter. The film developed for 24 h before being scanned with an Epson 10000XL (Seiko Epson Corporation, Suwa, Nagano, Japan) flatbed scanner. The calibration curve was created using Radiological Imaging Technology (RIT) (Radiological Imaging Technology, INC., Colorado Springs, CO) software. As EBT‐XD film exhibits minimal energy dependence, maximum variation of 1.5% in net optical density, the same calibration curve was used for the analysis of all EBT‐XD measurements.^13^

The EBT‐XD film was placed on a 15 cm solid water at depth of 6.5 cm for treatment plan delivery. A Standard Imaging A1SL ion chamber was placed at 7.5 cm depth in the solid water phantom for point dose verification. The gantry and collimator rotated as was planned in the TPS while the treatment couch was held fixed for the film delivery. After plan delivery, the EBT‐XD film was placed in a black film envelope to prevent ambient light contamination.

**FIGURE 1:**
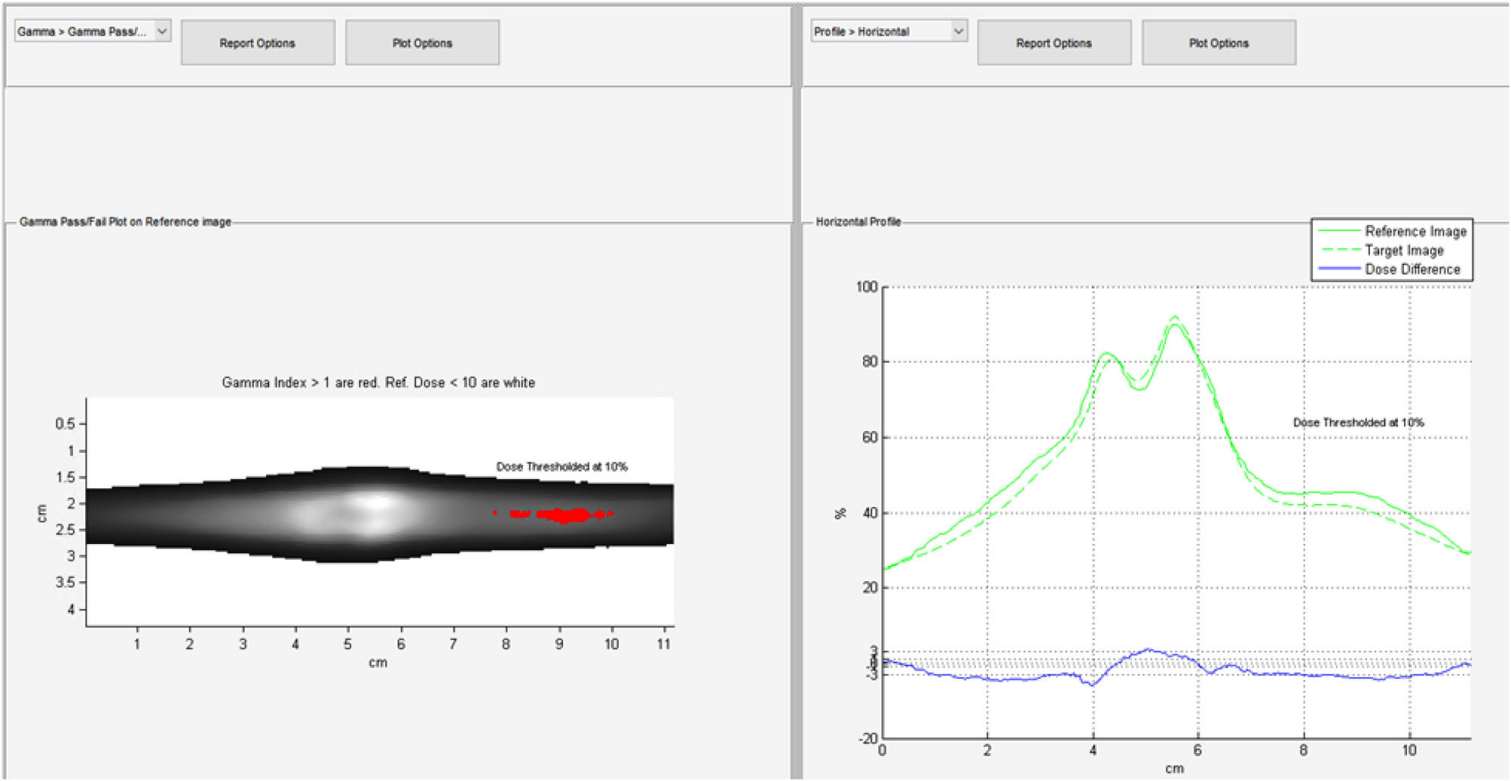
Measured dose distribution using EBT‐XD film in RIT software.

The irradiated film was scanned 24 h post exposure using an Epson 10000XL (Seiko Epson Corporation, Suwa, Nagano, Japan) flatbed scanner using the triple channel method, 72 dpi resolution (corresponding to 0.35 mm pixel spacing), and no color corrections. The film was handled in accordance with AAPM TG‐55 and TG‐235 to maintain the integrity of the film analysis and reduce lateral response artifacts.^11^, ^12^, ^13^ Scanned films were imported into RIT software as a reference image for analysis and the film calibration curve was applied. Subsequently, the calibrated film image was registered to the TPS calculated dose profile using 1 mm dose grid resolution as shown in Figure 1. The film was normalized to the global image maximum and the registered dose profiles were analyzed using the gamma test at 3%/3 mm, 2%/2 mm, 2%/1 mm, and 1%/1 mm using a 10% dose threshold.

#### 2.2.2 IBA Matrixx Resolution

The IBA Matrixx Resolution is a 25.3 × 25.3 cm^2^ 2D ion chamber array with 6.5 mm center‐to‐center detector spacing. There are 1521 vented parallel plate ion chambers on the array. Each ion chamber is 3.2 mm in diameter, 2 mm in height, and 12 mm^3^ in active volume. The bias voltage applied to each chamber is 500 ± 10 Volts.^14^

A CT plan of the IBA Matrixx Resolution was collected and exported to the Eclipse TPS to be used as a PSQA phantom. The Matrixx mini phantom and the detector absorber were assigned a density of 1.05 g/cm^3^ according the Matrixx manual. Additionally, the treatment couch and rails were included in the phantom so that the attenuation could be accounted for.

An array calibration was created for 6 MV to be used for 6 MV and 6 MV FFF beams and 10 MV to be used for 10 MV and 10 MV FFF beams. Dose calibration was also created for 6 MV, 6 MV FFF, 10 MV, and 10 MV FFF. At the time of each delivery session, the provided Gantry Angle Sensor (GAS) was attached to the gantry and paired with the myQA software. The GAS provides the current gantry angle to the myQA software for that measurement can be corrected for the angular dependence of the ion chamber array. Once, the measurement is collected, the TPS and measured profiles are registered and analyzed using the gamma test at 3%/3 mm, 2%/2 mm, 2%/1 mm, and 1%/1 mm.

#### 2.2.3 SNC ArcCHECK

The Sun Nuclear ArcCHECK is a helical array of n‐Si diodes that are spaced 1 cm apart and are placed 3 cm deep. The n‐Si diodes are 0.8 × 0.8 mm^2^ in size and are contained in layers of poly methyl methacrylate (PMMA). The active length of the detector is 27 cm and has a radius of 13.5 cm. The helical arrangement of the diodes allows each detector to measure entrance and exit dose to reconstruct the dose distribution. The center of the detector has a PMMA cavity plug in place to create a uniform density throughout the detector.^15^

In the TPS, the ArcCHECK virtual CT provided by Sun Nuclear was used and the density of the ArcCHECK body was assigned a density of approximately 1.2 g/cm^3^ and rods were assigned a density of 1.4 g/cm^3^. The treatment unit couch was also included to account for any attenuation of the treatment couch and rails.

QA plans were calculated using the native dose grid resolution of the treatment plan and exported to SNC Patient software for measurement. Dose calibration files were created for 6 MV, 6 MV FFF, 10 MV, and 10 MV FFF. Additionally, an array calibration was performed for 6 MV, to be used for 6 MV and 6 MV FFF deliveries, and 10 MV, to be used for 10 MV and 10 MV FFF deliveries. A 3D dose file is imported into the SNC Patient software and unraveled into a 2D form to be used as the reference profile in the gamma analysis.

#### 2.2.4 Varian DMI EPID

EPIDs were developed as an improvement conventional portal imaging using film cassettes. Image processing and display was made near instantaneous using the digital data collection of EPIDs. Additionally, errors resulting from dosimeter setup errors and angular dependence were reduced by the EPID being held in place at the defined source‐imager position that was perpendicular to the incident beam at all times. The Varian aS1200 Digital Megavolt Imager (DMI) EPID is a 43 × 43 cm^2^ array of amorphous‐Silicon (a‐Si) photodiode flat panel detector as the active layer. The design includes a 1 mm thick Cu build up layer, a phosphor screen scintillation layer made of gadolinium oxysulfide, an a‐Si photodiode read out layer, and a 4 mm thick backscatter layer made from Al and Pb plates. The pixel resolution is 1280 × 1280 pixels and each pixel has an approximate active area of 0.113 mm^2^. The DMI panel does support FFF beams with a high dose rate and can withstand up to 7000 cGy/Min without saturation effects.^16^, ^17^

Portal dosimetry plans were created and delivered using 100 cm source‐imager‐distance, couch rotation was fixed at 0 degrees (no rotation), but all gantry and collimator rotation settings were maintained for all treatment plans. Prior to data collection, the EPID panel was recalibrated in Service mode and a previously QA plan was delivered for verification. The collected portal dosimetry images were analyzed using a 10% dose threshold and MLC CIAO + 1 cm region of interest in the Portal Dosimetry workspace in Eclipse.

#### 2.2.5 SNC SRS MapCHECK

The SNC SRS MapCHECK is a 2D array consisting of 1013 diodes that are space 2.47 mm apart, center to center. The active area of each diode is 0.48 × 0.48 cm^2^ and an active volume of 0.007 mm^3^ and a sensitivity of 15 nC/Gy. The total active measurement area of the detector is 7.7 × 7.7 cm^2^. According to the specifications, the device can handle the maximum repetition rate of 3400 MU/min, which exceeds typical values at isocenter for the FFF 6 or 10 MV radiosurgical beams.

**FIGURE 2:**
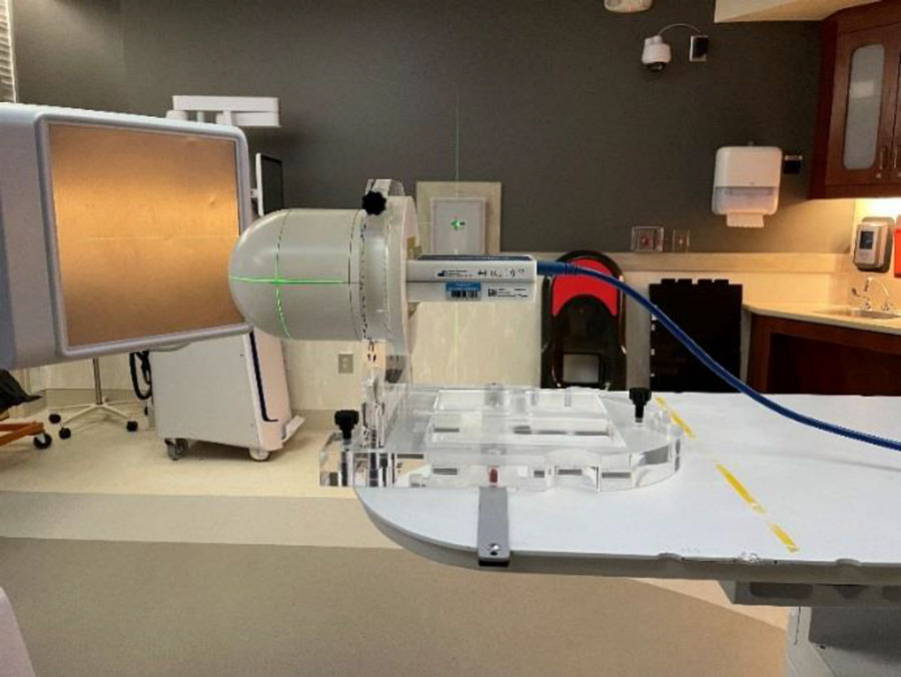
The SNC SRS MapCHECK inserted into the stereophan. The stereophan is indexed on the treatment couch such that the attenuation of the couch does not affect the detector measurement.

The SRS MapCHECK is designed to be inserted into the Sun Nuclear StereoPHAN as shown in Figure 2. The Stereophan system is cylindrical with one rounded end to approximate the shape of a patient's head. The body of the Stereophan is made from PMMA and two 2.2 cm thick PMMA plates are inserted above and below the SRS MapCHECK to provide buildup and backscatter to the detectors.^18^, ^19^, ^20^ The SRS MapCHECK was inserted into the Stereophan and was scanned on the CT using 1 mm slice thickness and exported to Eclipse. In Eclipse, the Stereophan body was delineated and assigned a density of 1.2 g/cm^3^. No treatment couch was included in the phantom model because the Stereophan is indexed so that the couch does not attenuate the beam during delivery.

QA plans were calculated 1 mm dose grid resolution of the treatment plan and exported to SNC Patient software for measurement. Dose calibration files were created for 6 MV, 6 MV FFF, 10 MV, and 10 MV FFF. Additionally, an array calibration was performed for 6 MV, to be used for 6 MV and 6 MV FFF deliveries, and 10 MV, to be used for 10 MV and 10 MV FFF deliveries.

#### 2.2.6 IBA myQA SRS

The IBA myQA SRS detector is a 2D array of monolithic solid‐state semiconductor detector that is placed between two layers of acrylonitrile butadiene styrene (ABS) (physical density of 1.05 g/cm^3^). The array consists of 300 × 350 pixels with 0.4 mm detector spacing across the entire 120 × 140 mm^2^ active area. The solid‐state detector is energy, dose rate, and angular dependent.^14^

The myQA SRS phantom with the myQA SRS inserted was scanned on the CT using 1 mm slice thickness and exported to Eclipse. In Eclipse, the myQA SRS phantom body and detector housing were delineated and assigned a density of 1.04 g/cm^3^. The active area of the detector was assigned a density of 1.0 g/cm^3^. The myQA SRS phantom did not index to the end of the table to prevent table attenuation so the treatment unit couch was also included to account for any attenuation of the treatment couch and rails.

QA plans were calculated using the native dose grid resolution of the treatment plan and exported to the myQA software for measurement. Dose calibration files were created for 6 MV, 6 MV FFF, 10 MV, and 10 MV FFF at 70% of the maximum dose rate of the plan. The 70% maximum dose rate was recommended by the manufacturer based on early testing observations of dose rate dependency. However, this information has not been published at the time of this writing. Additionally, a factory array calibration provided by IBA was utilized for all measurements based on the recommendation from the manufacturer.

**FIGURE 3:**
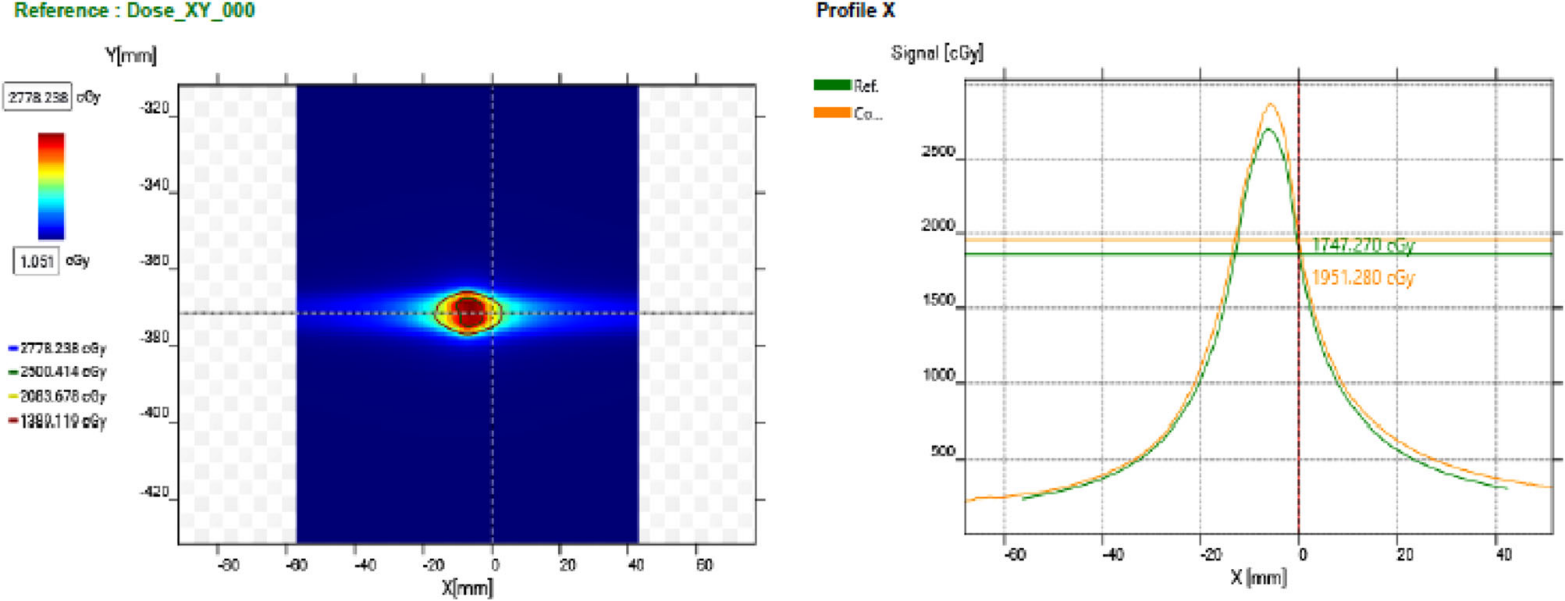
Measured dose distribution of 0.43 cc target from the IBA myQA SRS device. On the right, the measured crossline profile (green) is superimposed onto the calculated crossline profile (yellow) for the gamma analysis.

To account for angular dependency of the myQA SRS detector, a wireless GAS was placed on the gantry perpendicular to the plane of gantry rotation as shown in. During measurement the myQA SRS detector is placed in the myQA SRS phantom made from ABS and has a diameter of 19 cm. Collected dose profiles were registered to the 2D dose profile from Eclipse TPS and analyzed as shown in Figure 3.

#### 2.2.7 Gamma analysis comparison

Each plan was delivered onto all PSQA devices according to the methods described in the detector sections. The results were analyzed at 3%/3 mm, 2%/2 mm, 2%/1 mm, and 1%/1 mm gamma criteria and compared across devices for each treatment plan. During data collection, plans with passing rates of 90% or greater were considered passing based of the AAPM TG‐218 action limit.^21^ From this data, the device averages of each gamma criteria were collected for comparison across devices. The dependence on modulation factor, defined as the ratio of total plan MU to the prescribed dose per day, and target size were also investigated for correlation to gamma passing rates for each device.

#### 2.2.8 MLC position error detection

**FIGURE 4:**
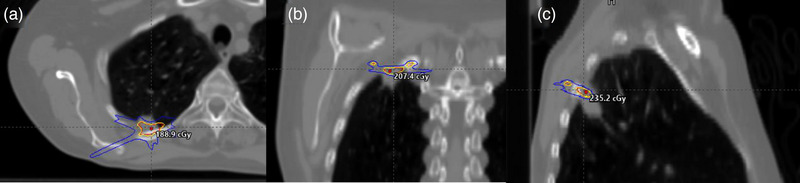
Axial (a), coronal (b), and sagittal (c) views of the dose difference composite of an error free lung SBRT plan and the plan with a 2 mm lagging MLC leaf. The max point in the dose difference on the composite plan is 259.4 cGy and the blue, yellow, and red isodose lines are 50, 100, and 110 cGy, respectively.

**FIGURE 5:**
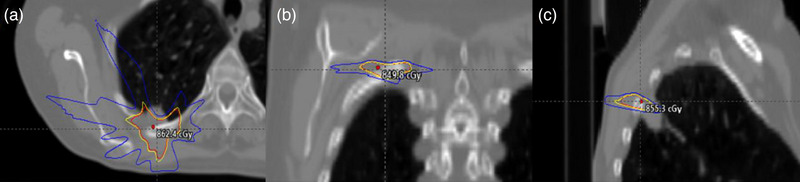
Axial (a), coronal (b), and sagittal (c) views of the dose difference composite of an error free lung SBRT plan and the plan with a stuck MLC leaf. The max point in the dose difference on the composite plan is 913.8 cGy and the blue, yellow, and red isodose lines are 125, 250, and 275 cGy, respectively.

MLC errors can lead to clinically significant changes in a dose distribution. Ten of the forty VMAT SBRT/SRS treatment plans were created with 6 MV FFF or 10 MV FFF beam energies using Eclipse 15.6. A MATLAB script was used to create two MLC positioning error scenarios from the original treatment plan: an MLC near central axis (CAX) that is lagging by 2 mm and an MLC near CAX that is stuck outside of the field. Figure 4 shows the dose difference composite of a lagging MLC plan and the original plan with a maximum dose of 259.6 cGy. Figure 5 shows the dose difference composite of a stuck MLC plan and the original plan with a maximum dose of 913.8 cGy. The original plan and both error plans were delivered on EBT‐XD, a Matrixx Resolution, ArcCHECK, and a myQA SRS. Each plan was analyzed with 3%/3 mm, 2%/2 mm, 2%/1 mm, and 1%/1 mm gamma criteria. The difference in gamma pass rates from the original plan to each error plan for each criteria were calculated and averaged.

## 3 RESULTS

High‐resolution detectors more reliably measured dose distributions than the lower‐resolution detectors when comparing the average gamma passing rate for each gamma criteria. A summary of the average gamma analysis results across all 40 plans can be found in Table 1.

**TABLE 1:** Average gamma results for 40 plans on each device.

	EBT‐XD Film	Matrixx Resolution	ArcCHECK	DMI EPID	SRS MapCHECK	myQA SRS
3%/3 mm	99.34%	96.70%	96.43%	99.30%	99.06%	99.71%
2%/2 mm	96.70%	89.77%	90.20%	95.93%	96.76%	97.91%
2%/1 mm	90.54%	76.51%	79.52%	78.22%	93.29%	92.55%
1%/1 mm	79.00%	64.71%	69.20%	71.57%	84.61%	84.31%

On average, lower‐resolution detectors did not correctly identify the changes in the dose distributions while high‐resolution detectors were able to distinguish the differences. The average gamma index difference of each detector for the 2 mm lagging MLC error plans can be found in Table 2.

**TABLE 2:** Lagging leaf average gamma index differences.

Lagging leaf avg. Gamma index difference				
Gamma criteria	3%/3 mm	2%/2 mm	2%/1 mm	1%/1 mm
Film	1.66%	4.94%	10.44%	13.83%
Matrixx Resolution	−0.51%	−0.33%	−1.98%	−2.68%
ArcCHECK	0.78%	−0.53%	−0.34%	1.69%
myQA SRS	−0.02%	0.12%	1.34%	1.73%

Lower‐ and high‐resolution detectors did correctly identify the changes in dose distribution caused by stuck MLC leaf. The average gamma index difference of each detector for the stuck MLC error plans can be found in Table 3.

**TABLE 3:** Stuck leaf average gamma index differences.

Stuck leaf avg. Gamma index difference				
Gamma criteria	3%/3 mm	2%/2 mm	2%/1 mm	1%/1 mm
Film	3.99%	9.71%	19.24%	22.86%
Matrixx Resolution	0.09%	1.44%	1.37%	2.04%
ArcCHECK	1.43%	3.16%	5.13%	6.87%
myQA SRS	0.18%	1.77%	6.16%	5.97%

Additionally, when comparing passing rates to modulation factor and target volume, no correlation could be determined for any of the patient specific QA devices.

The summary of detector dependencies can be found in Table 4.

**TABLE 4:** Summary of detector dependencies.

Dependency						
	Target volume	Modulation	Angular	Dose rate	Energy	Temperature and pressure
EBT‐XD	–	–	–	–	–	–
Matrixx Resolution	–	–	–	–	–	×
ArcCHECK	–	–	×	×	×	–
DMI EPID	–	–	–	–	×	–
SRS MapCHECK	–	–	×	×	×	–
myQA SRS	–	–	×	×	×	–

### 3.1 EBT‐XD film

EBT‐XD films average gamma analysis were greater than 90% for 3%/3 mm, 2%/2 mm, and 2%/1 mm. EBT‐XD film was able to detect changes in measured dose distribution for both lagging and stuck MLC scenarios at all gamma analysis criteria.

### 3.2 IBA Matrixx Resolution

All plans that were analyzed at 3%/3 mm passed at 90% or greater. Average gamma results agreed with a film within 3% at 3%/3 mm criteria, but this agreement disappeared at more strict criteria.

### 3.3 SNC ArcCHECK

Similarly, to the IBA Matrixx Resolution, ArcCHECK did agree with film results within 3% at 3%/3 mm, but the agreement deviated rapidly as criteria became stricter. The Sun Nuclear ArcCHECK performed similarly to that of the IBA Matrixx Resolution in the Gamma Analysis comparison and the MLC error detection.

The SNC ArcCHECK average differences in gamma passing rates were 0.78%, −0.53%, −0.34%, and 1.69% for the 2 mm lag plan for 3%/3 mm, 2%/2 mm, 2%/1 mm, and 1%/1 mm gamma criteria, respectively. For the stuck MLC leaf plans, the average differences in gamma passing rates were 1.43%, 3.16%, 5.13%, and 6.87% for 3%/3 mm, 2%/2 mm, 2%/1 mm, and 1%/1 mm gamma criteria, respectively. The ArcCHECK was able to detect the changes in dose distribution of the stuck MLC leaf plans for all gamma criteria, but did not detect the error in MLC position for 2 mm lag plans.

### 3.4 Varian DMI EPID

These results agree with a film within 2% for 3%/3 mm and 2%/2 mm, but begin to deviate for 2%/1 mm and 1%/1 mm. Interestingly, 35 out of 40 plans passed strongly above 90% at 2%/2 mm, but only 3 out of 40 plans passed above 90% for 2%/1 mm.

Unfortunately, although the error plans were delivered using the DMI EPID, no data could be collected due to the nature of how portal dosimetry fluence is calculated. Furthermore, using a third‐party software to analyze the results was considered, but a known limitation in Eclipse v. 15.6 prevented the accurate export of composite fluence distribution for analysis.

### 3.5 SNC SRS MapCHECK

The SRS MapCHECK results agreed with EBT‐XD results within 1% for 3%/3 mm and 2%/2 mm and within 3% at 2%/1 mm. Both 2%/1 mm and 1%/1 mm outperformed the results of EBT‐XD by 2.75% and 5.61%, respectively. MLC positioning error tests were not performed on the SRS MapCHECK due to device availability at the time of data collection so no data was collected for these tests.

### 3.6 IBA myQA SRS

The myQA SRS performed very similarly to the SNC SRS MapCHECK. In comparison to EBT‐XD film, there is good agreement within 2.5% at 3%/3 mm and 2%/2 mm, and 2%/1 mm for the average gamma passing rates. At 1%/1 mm, the myQA SRS average gamma passing rate was 5.31% higher than EBT‐XD film.

The IBA myQA SRS detector average difference in gamma passing were −0.02%, 0.12%, 1.34%, and 1.73% for the 2 mm lag plan for 3%/3 mm, 2%/2 mm, 2%/1 mm, and 1%/1 mm gamma criteria, respectively. The IBA myQA SRS did detect the changes in dose distribution when examining the collected profiles, however, the changes were not significant enough to change the gamma index for any of the 2 mm MLC plans. At 2%/2 mm only 3 out of 10 plans resulted in a significant change to the gamma index while 1 out of 10 actually increased the gamma index. Significant detection of the 2 mm MLC position error became apparent at 2%/1 mm.

The stuck MLC plan average gamma index differences were 0.18%, 1.77%, 6.16%, and 5.97% for 3%/3 mm, 2%/2 mm, 2%/1 mm, and 1%/1 mm gamma criteria, respectively. At 3%/3 mm the changes for gamma index were still small, but the gamma index changes improved as stricter criteria were used.

## 4 DISCUSSION

As stated in the Results section, EBT‐XD films had average gamma analysis passing rates of 99.34%, 96.70%, 90.54%, and 79.00% for 3%/3 mm, 2%/2 mm, 2%/1 mm, and 1%/1 mm gamma criteria, respectively. EBT‐XD has superior spatial resolution compared to the SRS MapCHECK and myQA SRS devices and has no significant dependence on energy or dose rate. However, EBT‐XD underperformed at 1%/1 mm gamma criteria in this study compared to the SRS MapCHECK and myQA SRS. A possible reason for this discrepancy could be that film analysis procedures could be prone to systematic errors. For example, Lynch et al. investigated the variations in optical density reading by flatbed scanners based on film position and angular rotation of the film when it was placed on the scanner. They reported nonuniformity of up to 17% along the scanline and 7% perpendicularly to the scanline for films that were uniformly irradiated and scanned at different locations on the flatbed scanner. The films were rescanned at different angular rotations in 45‐degree increments. An 8% variation was reported for the film irradiated with 300 cGy. Leveraging the spatial resolution of EBT‐XD film requires a strict methodology to reduce the sources of error in the dose distribution analysis.^22^

### 4.1 Measured profile discrepancies

As shown in Table 1, the gamma analysis results of the IBA Matrixx Resolution and the SNC ArcCHECK decrease with more stringent gamma criteria more rapidly when compared to EBT‐XD film, SRS MapCHECK, and the myQA SRS device. Detector resolution plays a dominant role in a detectors ability to measure a dose distribution with fidelity as the complexity of the distribution increases. Therefore, the detector spacing and spatial resolution of the ArcCHECK and Matrixx Resolution systems limits their ability to measure complex dose distribution resulting in a decline in gamma analysis results which is discussed below.

**FIGURE 6:**
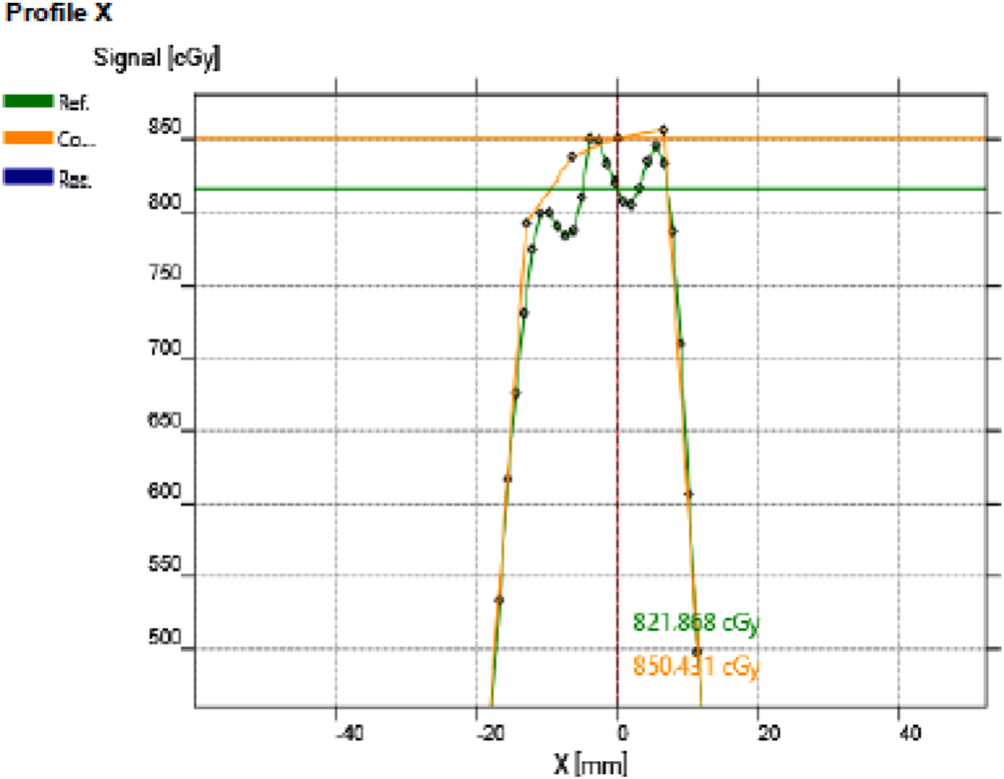
Undersampling of a 10.19 cc target. The measured profile (yellow) appears undersampled compared to the calculated profile (green).

The IBA Matrixx Resolution appeared to lack the necessary resolution to capture the complexity of the dose distribution of some plans. Figure 6 shows the measured (yellow) and calculated (green) profiles of a 10.19 cc target that passed at 95.9%, but the profile is undersampled and possible volume averaging is being exhibited.

**FIGURE 7:**
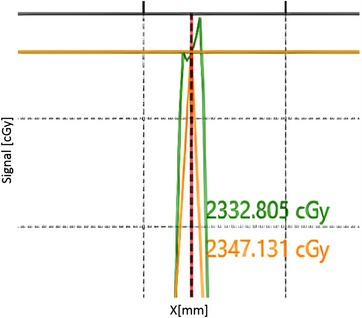
The measured (yellow) profile under sampling of a 0.43 cc target passing at 3%/3 mm compared to the calculated (green) profile.

Additionally, Figure 7 shows a 0.43cc target that passes 3%/3 mm gamma criteria at 96.2%. However, when comparing the dose distribution, it is observed that the measured profile does not satisfactorily match the calculated profile despite the 3%/3 mm passing rate.

The IBA Matrixx Resolution average differences in gamma passing rates were −0.51%, −0.33%, −1.98%, −2.68% for the 2 mm lag plan and 0.09%, 1.44%, 1.37%, and 2.04% for the stuck MLC plan for 3%/3 mm, 2%/2 mm, 2%/1 mm, and 1%/1 mm gamma criteria, respectively. The IBA Matrixx Resolution was unable to correctly detect the dose distribution error caused by a leaf lagging by 2 mm. In contrast, the Matrixx correctly detected differences in the dose distribution for a leaf that was stuck although this more consistently occurred at 2%/2 mm and stricter gamma criteria.

**FIGURE 8:**
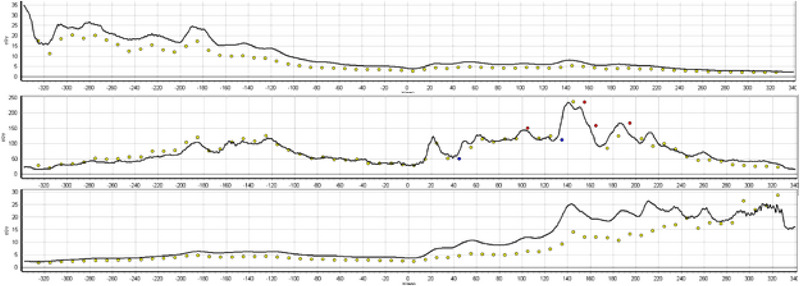
Three rows of diodes from the measurement of a small SRS plan. The top image is the row above the target, the middle image is the row of diodes that received direct beam, and the bottom image is the row of diodes below the target.

The Sun Nuclear ArcCHECK uses diodes to provide good spatial resolution and it is not prone to volume averaging like ion chambers. The body of the ArcCHECK is designed as a cylinder so that these diodes are always perpendicular to the angle of delivery, thus improving the angular dependence of the detector. The diodes of the ArcCHECK are spaced by 10 mm so for very small targets, it is possible that only one row of diodes may be within the field, limiting the data that is collected. Figure 8 illustrates that only one row of diodes received direct irradiation. Diodes outside of the direct beam also exhibit under response due to the small dose in that region.

### 4.2 Multi‐target plans

There were three multi‐target plans included in this study: two cranial cases with two targets each and one lung case with two targets. Multi‐target plans can be challenging for detectors due to the complex fluence that is generated by the LINAC. Additionally, the targets may not be positioned on the detector plane so the detector measures the dose between the targets which may not be as clinically significant as the dose inside the targets.

**FIGURE 9:**
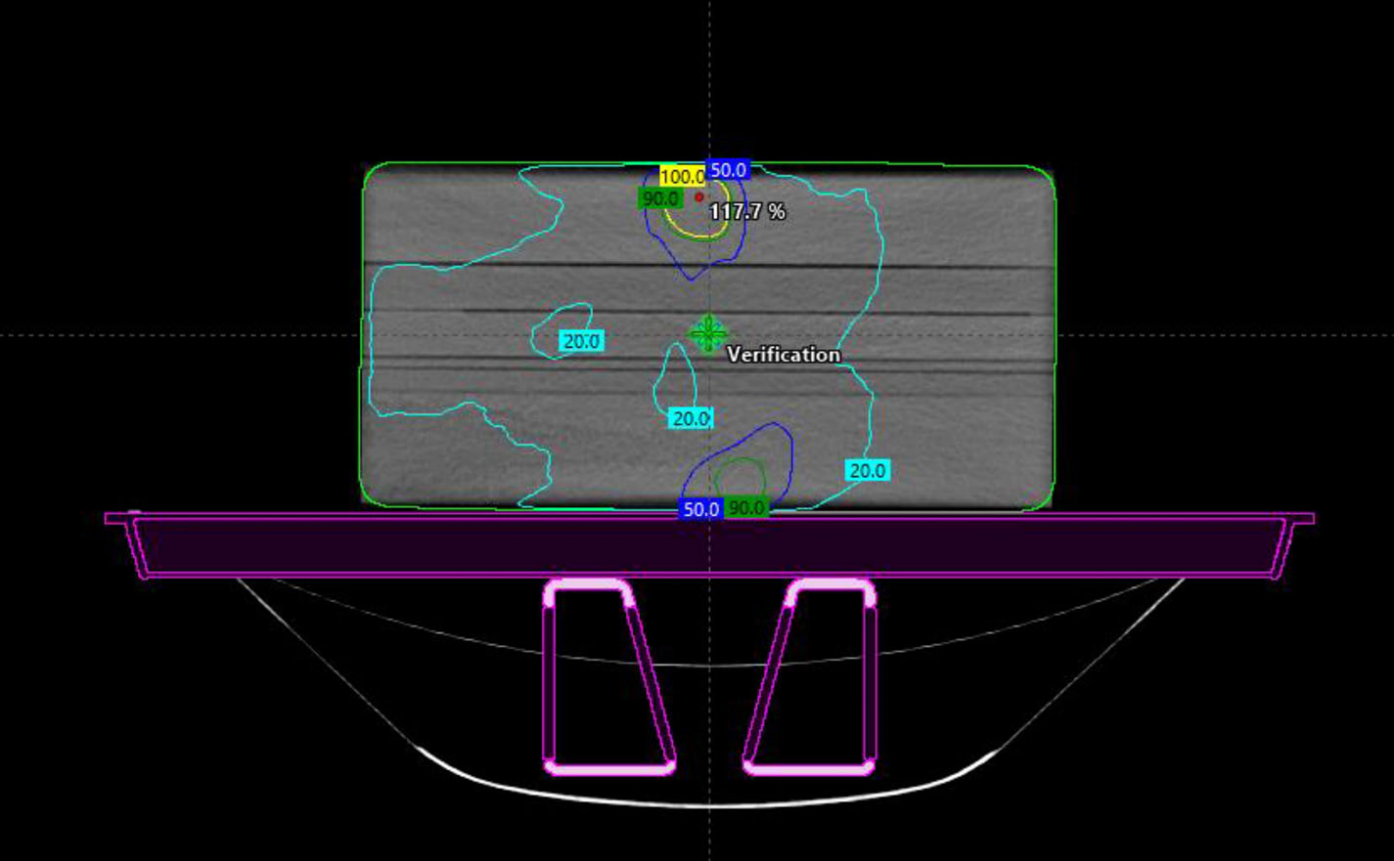
Multi‐target lung SBRT plan calculated on 15 cm solid water film phantom. Neither target is on the same plane as the EBT‐XD film.

EBT‐XD film multi‐target plans analysis results were below average for each gamma criteria in comparison to the overall EBT‐XD gamma index averages. The worst plan was the lung case where the 3%/3 mm gamma index failed at 88.73%. None of the targets were positioned on the same plane as the film location within the phantom so the dose collected on each of these plans was dose in between the targets. The IBA Matrixx resolution had similar results on the multi‐target plans as EBT‐XD, however, all plans passed greater than 90% at 3%/3 mm. Figure 9 shows a multi‐target lung plan calculated on the 15 cm solid water phantom.

The ArcCHECK and DMI EPID were the only two detectors that were able to directly measure the target doses due to their design. Two of the three plans passed at 2%/2 mm for the ArcCHECK and all plans passed at 2%/2 mm for the DMI EPID. Neither detector had any plans pass at 2%/1 mm or 1%/1 mm.

In contrast, SNC SRS MapCHECK and myQA SRS performed well for the multi‐target plans with all plans passing at 3%/3 mm, 2%/2 mm, and 2%/1 mm. For the SNC SRS MapCHECK and myQA SRS, the targets were not located on the same plane as the detector. Despite this, all plans passed above 90% for the 2%/1 mm and one plan passed at 1%/1 mm for the SRS MapCHECK. Overall, the SRS MapCHECK and myQA SRS detectors were able to consistently provide high passing rates at strict gamma criteria.

The gamma analysis comparison compared 40 SRT plans at various gamma criteria. Five of the six detectors were 2D arrays of detectors some of which required special phantoms for treatment delivery such as the Sun Nuclear Stereophan. A limitation of this study is the characteristics of each phantom such as the material and design were not investigated to determine the role these phantoms may have had in the measurement for each detector.

Additionally, each detector was oriented in the coronal plane during delivery. The Varian aS1200 EPID which rotates with the gantry and is always perpendicular to the incident beam and the ArcCHECK which is cylindrical in design, also mitigating the angular dependency of the device are both exceptions to the detector orientation. Fixing the detector orientation to the coronal plane did limit the ability of this study to provide data on some of the more useful functions of some of these detectors. For example, the IBA myQA SRS and Sun Nuclear SRS MapCHECK are both capable of rotation along the coronal axis within their phantoms. This ability is particularly useful in the multi‐target setting so the detector plane can be rotated to be coplanar with a target. Therefore, if the targets were not coplanar with the detector, the detector was only measuring the fluence between the targets and not the high dose areas that are of more clinical interest similar to the solid water phantom used for EBT‐XD film in Figure 9.

## 5 CONCLUSION

A comparison of commercial QA devices to the film dosimetry method was evaluated. For the gamma analysis comparison, all detectors have greater than 95% passing rates at 3%/3 mm gamma criteria. However, passing rates decrease rapidly for ArcCHECK and the Matrixx as criteria became more strict. In contrast, EBT‐XD film, SNC SRS MapCHECK, and IBA myQA SRS passing rates do not decline as rapidly and maintain greater than 90% passing rate at 2%/1 mm and greater than 79.00% at 1%/1 mm.

For the MLC position error tests, a negative average gamma difference indicated that the plans with the errors present passed better than the original plan. In contrast, a positive average gamma difference indicates that on average, the error plan did not pass as high as the original, error‐free plan. High‐resolution detectors performed better for both types of scenarios for MLC positioning errors. Changes in passing rate became most apparent using the 2%/1 mm gamma criteria.

SRT plans require high accuracy in all aspects in treatment delivery. To ensure the fidelity of the dose distribution that will be delivered to the patient, stricter gamma analysis criteria are required. In our analysis, 2%/1 mm would be the best choice. Therefore, EBT‐XD film, IBA myQA SRS, SNC SRS MapCHECK, or similar high‐resolution detector that is a capable of accurately measuring the complex dose distributions is recommended for the QA of SRT plans.

## AUTHOR CONTRIBUTIONS

All authors in this manuscript in one more of the following ways: study design and development, data collection and analysis, results and conclusion synthesis, and editing/revising.

## CONFLICT OF INTEREST STATEMENT

The authors declare no conflicts of interest.
